# Lateral tibial condyle reconstruction by pedicled vascularized fibular head graft: long-term result

**DOI:** 10.1007/s11751-011-0108-1

**Published:** 2011-05-17

**Authors:** Syed Kamran Ahmed, Boris Kwok Keung Fung, Wing Yuk Ip, Shew Ping Chow

**Affiliations:** Division of Hand and Foot Surgery, Department of Orthopaedics and Traumatology, Queen Mary Hospital, University of Hong Kong, 102, Pok Fu Lam Road, Pokfulam, Hong Kong

**Keywords:** Lateral tibial condyle reconstruction, Pedicled vascularized fibular graft, Aggressive giant cell tumours

## Abstract

The technique of pedicled vascularized fibular graft for lateral tibial condyle reconstruction after en bloc resection of aggressive giant cell tumours was described by SP Chow et al. Early follow-up of two patients was presented in 1986. We present the 25 years follow-up of one patient with a literature review of alternative present day treatment options. The patient maintained community ambulant status despite developing late stage osteoarthritis. Although this procedure is performed rarely, it remains an alternative to the more sophisticated treatment options making it a useful method in centres with limited facilities and expertise.

## Background

En bloc resection of aggressive giant cell tumours of bone has been shown to produce better local control of disease [1]. Numerous options are available for defect reconstruction, each with a different set of complications which remains a matter of concern.

The senior author developed a technique of pedicled vascularized fibular graft using fibular head as a replacement of the lateral tibial defect after en bloc resection of a giant cell tumour in 1982. Early follow-up of two cases was published in 1986 [2]. The long-term problems especially tumour recurrence and disabling knee arthritis remained a matter of concern.

Although the literature has supported the use of pedicled fibula for tibial defect reconstruction in the years to follow, no further reports of lateral tibial condyle reconstruction with the vascularized pedicled fibular head graft have been cited so far.

We present a 25 years follow-up of one such case and a literature review of current alternative treatment options available.

## Details of surgical procedure

### Applied anatomy

**Fig. 1:**
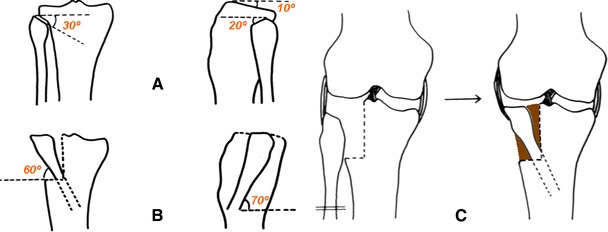
Applied anatomy. **a** The normal anatomy and relationship of the proximal tibia and fibula. **b** The AP and lateral diagrammatic views after the fibula transfer. **c** Surgical technique diagram

The blood supply of the upper fibula is derived from nutrient vessels entering the shaft at the junction of the upper and middle third and by periosteal vessels. During mobilization, there should be minimal dissection to preserve the periosteal blood supply. The articular surface of the proximal fibula faces medially and forms the proximal tibiofibular joint. As seen in an AP view, it slopes at an angle of 30° to the horizontal plane. As seen on the lateral view, there is also a 10° downward and posterior slope of the lateral tibial condyle, whereas the head of the fibula has an anterior slope of 20°. The fibula lies posterior to the central weight bearing portion of the lateral tibial condyle (Fig. 1a). In order to conform to the surfaces of the femoral condyle in full extension, the whole graft needs to be shifted anteriorly and fixed with the fibular head directed posteriorly and laterally (Fig. 1b). The medial displacement of the graft ensures lateral stability by tightening the lateral collateral ligament and the biceps femoris tendon (Fig. 1c).

### Surgical technique (Fig. 1a–c)

A longitudinal curvilinear incision is used for anterolateral approach. The common peroneal nerve is identified and saved. The fibula is sectioned slightly lower than the level of resected tibial condyle. The upper portion of the fibula is moved medially with its blood supply intact, preserving the attachments of joint capsule, lateral collateral ligament and biceps femoris tendon. The fibular graft is then jammed into tibia and the fixation supplemented by a screw. The place between the graft and the remaining bone is packed with cancellous graft from the iliac crest. The wound is closed over suction drains. The average operating time is 2 h.

### Rehabilitation guidelines

A long leg cast is applied for 6 weeks, followed by hinged knee brace until clinical and radiographic evidence of bony union is seen, usually at 3 months.

## Case report

**Fig. 2:**
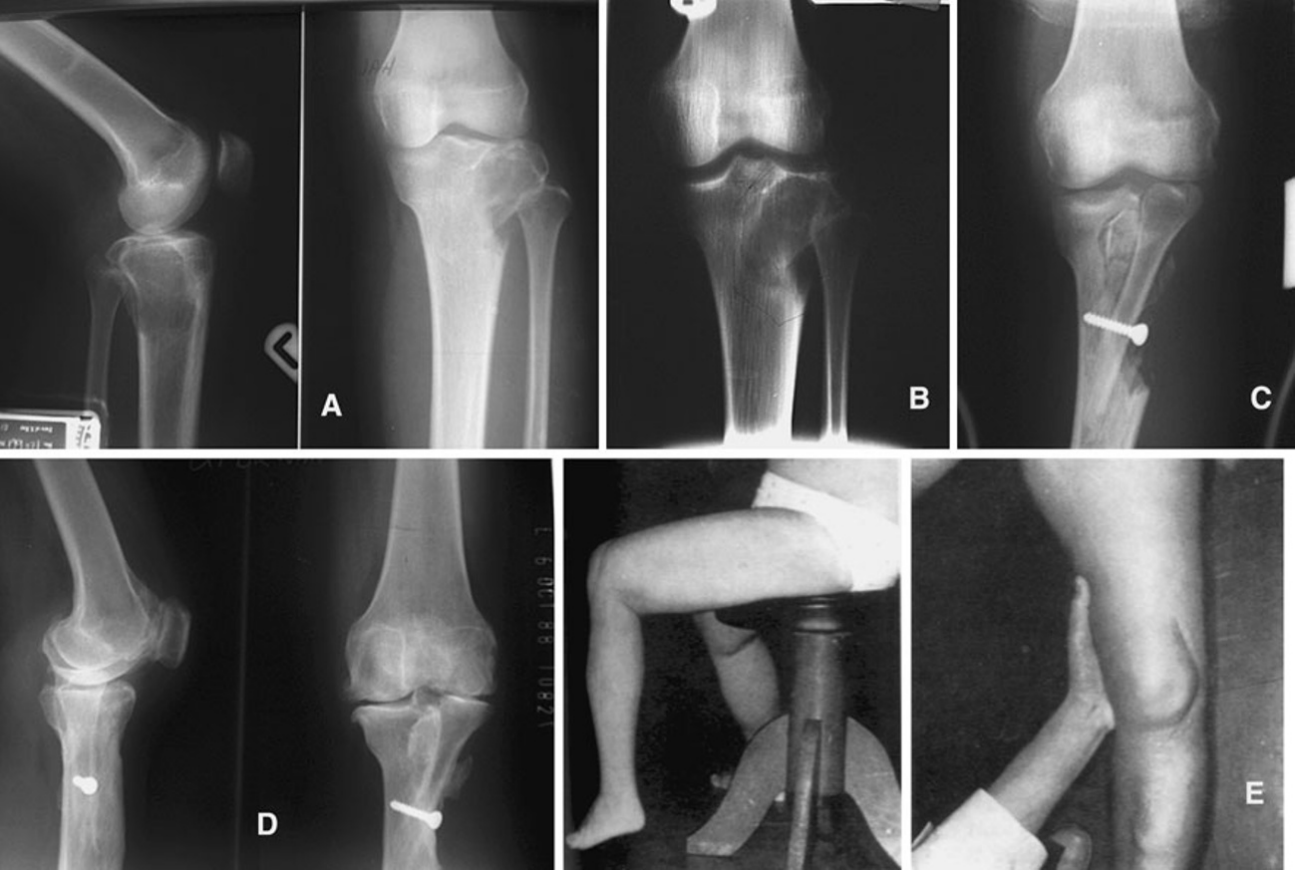
Early result. **a** Preoperative X-ray showing aggressive giant cell tumour of the lateral tibial condyle. **b** AP tomogram showing the extent of lesion and possible joint penetration. **c** Early healing at 12 weeks, patient still in brace. **d** X rays at 5 years showing well preserved joint surfaces despite early arthritic change. **e** Early clinical photograph at 2 years

A 22-year-old female presented with a history of left knee pain and swelling of 10 month duration. On examination, there was mild swelling in the region of the lateral tibial condyle. The range of knee motion was from 0° to 120°. Plain radiographs revealed an eccentric, expansile and radiolucent lesion in the lateral tibial plateau reaching up to the joint line consistent with a giant cell tumour (Fig. 2a). A tomogram was performed which revealed a possible cortical breach along the joint line (Fig. 2b). An en bloc resection was performed, confirming the preoperative diagnosis, and reconstruction was performed as described. Full weight-bearing ambulation was started at 15 weeks (Fig. 2c). The patient maintained a good joint space at 5 years from surgery, and she was able to swim, play badminton and tennis without any discomfort (Fig. 2d). She maintained 0°–90° of knee motion and had a mild residual lateral laxity at 45° of knee flexion (Fig. 2e).

**Fig. 3:**
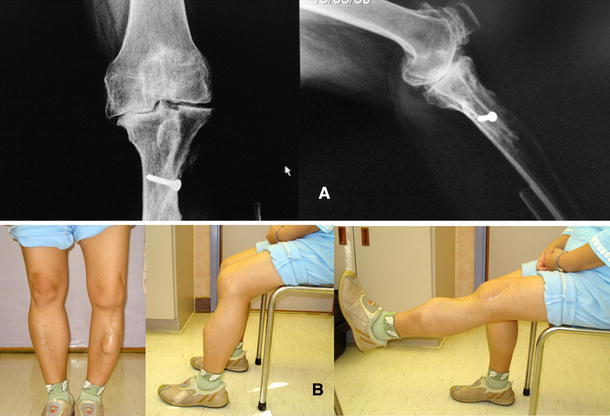
Twenty-five years result. **a** X-rays showing tri-compartmental osteoarthritis of the knee joint. **b** Clinical photographs at 25 years

Clinical follow-up at 25 years showed tri-compartmental osteoarthritis (Fig. 3a). She had a 10° fixed flexion contracture, with a range of knee motion from 10° to 70°. The tibia developed 08° of varus at the knee (Fig. 3b).

At 5 years, the Knee Society Score (KSS) was 83 and Knee Society Functional Assessment (KSFA) Score was 90. The 25 years follow-up KSS is 75, and the KSFA score is 70.

There has been no tumour recurrence. Occasional use of oral pain medications and activity modification was required. The patient had developed a stiff knee gait with time, but was satisfied with the final result of the procedure.

## Discussion

The use of fibular head autograft for joint surface replacement of the lateral tibial plateau was suggested by Jackson in 1972. A recent cadaver study has shown that the surface area of the fibular head is 3.12 cm^2^ whereas that of the central weight-bearing portion of the lateral tibial plateau is 3.64 cm^2^. The authors have objectively documented good results in five patients with severely comminuted lateral tibial condyle fractures treated with a fibular head autograft [3].

The same concept was adapted by Chow et al. for the reconstruction of the tibial condyle after en bloc resection of giant cell tumours, with preservation of the blood supply as a pedicled vascularized graft in 1982. A vascularized graft does not undergo resorption and creeping substitution. It rather progresses to rapid fusion and adapts to physiological load.

It is encouraging to have no tumour recurrence at 25 years follow-up. The gradual degeneration in the knee joint in our patient was anticipated initially, due to meniscus damage and joint laxity. Despite this fact, the patient remained fully active in full time employment at 25 years.

The present day trend in patients undergoing en bloc resection for aggressive giant cell tumours is the use of hemicondylar and total condylar osteoarticular allografts and allograft prosthetic composites [4]. Although encouraging results have been reported, appropriate size matching of the allograft, technically demanding reconstruction of the ligaments, tendons and capsule and above all the risk of tissue-transmitted diseases remains a concern. The reported 5 years survival and good results in major series are 73%. The articular cartilage deterioration is evident in as many as one third patients by 5 years [4].

Another well cited option is the reconstruction with vascularized patella [5]. The procedure has shown to delay the need for a future reconstructive procedure for approximately two decades. Apart from the development of osteoarthritis, the reported complication rates are high with a 50% re-operation rate [5]. Patellectomy has been shown to induce long-term disability due to thirty percent decrease in quadriceps strength.

The use of iliac crest graft as described by Lee HG in 1957 is an inferior option as it replaces joint surface with bone instead of cartilage.

Finally, the most popular method of intralesional excision, which is controversial for the treatment of aggressive giant cell tumours, is most suited for contained lesions [1]. However, with careful patient selection, it still remains the procedure of choice for most of the giant cell tumours around the knee.

We would like to stress the need of careful preoperative assessment of tumour spread by MRI and further by intraoperative frozen sections if doubt exists about the aggressiveness of the lesion.

The tissue banks and their regulations are still new to most of the developing nations.

Our experience shows that the pedicled vascularized fibular head transfer is a very promising option for selected young patients, especially in centres without the sophisticated facilities or expertise. It avoids the use of allograft, prosthesis and disabling knee arthrodesis in young patients, as well as providing sufficient bone stock for future reconstructive options.
